# Adolescents who are overweight or obese - the relevance of a social network to engaging in physical activity: a qualitative study

**DOI:** 10.1186/s12889-021-10727-7

**Published:** 2021-04-09

**Authors:** Ingeborg B. Skogen, Kjetil L. Høydal

**Affiliations:** Faculty of Arts and Physical Education, Department of Physical Education, Volda University College, Volda, Norway

**Keywords:** Obese, Overweight, Physical activity, Primary health care, Self-determination theory, Social network, Young people

## Abstract

**Background:**

Increased level of physical activity (PA) is one of the approaches offered by school health services in Norway for the prevention and treatment of children and adolescents with overweight and obesity. Research has found that young people with overweight and obesity tend to participate in less physical activity than youth of healthier weight. They also tend to perceive several barriers to PA at the environmental, interpersonal, and individual levels. This paper explores how adolescents’, who receive follow-up of weight management in primary health care, experience barriers to and facilitators for engaging in physical activity within their social networks.

**Methods:**

This is a qualitative study with an explorative design inspired by phenomenological ideas based on analysis of semi-structured interviews with 10 adolescents aged 13–18. Interviews were conducted with young people with overweight or obesity in weight-related follow-up in primary health care settings in various regions in Norway.

**Results:**

The results describe the barriers to and facilitators of PA in the adolescents’ social networks. The study reveals a strong emphasis on the importance of a social network for engaging in PA. According to these adolescents’ experiences of PA in their social networks, organizing PA groups that consist of similarly young people with overweight can contribute to an increased level of PA and help modify their negative perceptions of such activity.

**Conclusions:**

This study finds that young people with overweight and obesity place a strong emphasis on a social network where they feel equal to others if they are to engage in PA. The study suggests that School Health Nurses should establish a social network of adolescents in the same situation, in the form of organized activity groups for young people with overweight or obesity, rather than advice on increased participation in ordinary organized sports or individual exercising. This can facilitate an increase in the PA level among this group of young people.

**Supplementary Information:**

The online version contains supplementary material available at 10.1186/s12889-021-10727-7.

## Background

Overweight and obesity have become one of the world’s greatest public health challenges [1, 2]. Over the last decades, there has been a worldwide rising trend in children’s and adolescents’ body mass index (BMI), which now has plateaued in many high-income countries [3]. In Norway, among children aged 8 years, 13% of boys and 17% of girls are categorized as overweight or obese, while about 23% of 17-year-olds are overweight or obese [4]. In 2010, the Norwegian Directorate of Health introduced national clinical guidelines for the prevention, identification, and treatment of overweight and obesity in children and adolescents. The guidelines recommend follow-up or treatment of children and adolescents who have a BMI ≥ iso-BMI 25 based on standard definitions for child overweight and obesity worldwide (The International Obesity Task Force) [5], and after screening for other factors which can have an impact on overweight. Follow-up and treatment are optional for families and focuses on motivating changes in physical activity (PA) and diet. School health nurses (SHNs) are the key personnel delivering this work [6]. The effectiveness of interventions in health care settings is to a great degree dependent on people’s adherence to self-care programs and activities [7], notably recommendations for an increased level of PA, as discussed in this study. Poor adherence to such recommendations over time is a significant challenge for these types of interventions [7].

Increased level of PA is one of the approaches offered by school health services in Norway to tackle overweight and obesity. The general PA recommendation to achieve health benefits in children and adolescents is at least 60 min of moderate to vigorous exercise daily [8]. Many young people do not reach the recommended level of PA. Research has found that young people who are overweight or obese tend to participate in less physical activity than youths of healthier weight [9, 10].

Stankov, Olds, and Cargo [11] describe a range of barriers to PA participation among adolescents who are overweight or obese, at the environmental, interpersonal, and individual levels. Environmental and interpersonal circumstances may contribute to increased negative self-perceptions in these young people in school or generalized settings. Being an overweight in adolescence seems to predispose a young person to a higher level of victimization and difficulty in forming relationships with peers, which according to previous research are related to participation in PA [11]. A review of the influence of peers and friends on PA level among young people with overweight or obesity suggests that the presence of peers and friends could provide a social structure fostering motivation to be active. The importance of friendships in this context is confirmed in other research [12, 13]. In adolescence there is an increasing independence from family, and the social networks external to the family environment expands [14]. Research has shown that among youth at risk of physical inactivity (including youth with overweight), activity-related support from both parents and siblings, but also friends may be effective in promoting PA participation. Parents should be aware of the importance of peers and the role they can play in fostering adolescents’ interest in PA. To help foster support within the social network, it is proposed that parents could identify popular activities, organize activities that include a friend, or facilitate activity when adolescents spend time with friends [15]. In children and adolescents there is shown higher levels of PA when they are taken to places for activity by their parents and enrolled in organized activities [16, 17]. The probability of engaging in PA also increase when there is positive interactions, mutual affection, cooperation, and respect. It may be that adolescents who are overweight and/or obese do not respond positively to recommendations for an increase in PA level because they lack positive peer relations and they therefore have to perform PA on their own [18].

According to this, it is important to understand the barriers to PA and how adolescents perceive PA in their social networks in order to comprehend their motivation for (in)activity. The Self-Determination Theory (SDT) has proven useful in understanding behavioral motivation and the reasons for the adaptation and maintenance of behavior change, such as increased PA [19–21]. SDT is an organismic–dialectical metatheory, in which humans are seen as active, growth-oriented organisms and the framework is built upon the dialectical relation between people and the social environment. According to SDT, individuals try to satisfy three basic psychological needs: autonomy, competence, and relatedness; and the motivation for certain behaviors can be amotivation, extrinsic or autonomous, presented on a continuum. Autonomous motivation refers to actions performed for their own intrinsic purpose, such as enjoyment or challenge; at the other end of the continuum is controlled motivation, which refers to actions associated with extrinsic rewards [19, 20, 22]. Ng et al. [23] report that motivation and behavior with regard to weight management may be influenced by individuals’ perception of how supportive and controlling their important others are. Further, Ng et al. [24] indicate that the quality of interactions between individuals engaged in weight management and their important others matters in terms of predicting their psychological needs and well-being. Perceived support for autonomy from important others is related to the satisfaction of psychological needs, while controlling behavior is associated with thwarting such needs [24]. In interviews with adolescents participating in a 12-week internet-based intervention study [25], Sundar et al. found that adolescents with overweight or obesity’s positive experiences of participation in organized PA are related to the feeling of mastering an activity and knowing their teammates. The motivation to participate increases further if the activity is self-chosen. However, studies on adolescents who have not taken part in such interventions are notably absent [26].

To the best of our knowledge, no previous qualitative study has focused on adolescents with overweight or obesity who are only receiving follow-up in the primary health care setting, and the experiences of barriers and facilitators in their social network for engagement in PA. There is a lack of perspectives from young people with overweight or obesity on experiences with recommendations for an increase in PA level within their social network, as part of follow-up given in primary health care settings. The present study aims to explore how adolescents’, who receive follow-up of weight management in primary health care, experience barriers to and facilitators for engaging in physical activity within their social networks.

## Methods

### Design and method

To gain insight into the experiences of adolescents who are overweight or obese, an explorative design inspired by phenomenological ideas was chosen as the study aims to describe and understand how the world is perceived by the young people [27].

Semi-structured individual interviews were conducted to explore how adolescents perceive the barriers and facilitators in their social network for engaging in PA.

### Context

In Norway, school health nurses work at public health centers as part of the primary health care service for children and adolescents. Public health centers see all children from birth and monitor the child’s health and development. As part of the health and development monitoring, SHNs follow up school children and adolescents’ weight and height at mandatory measurements in grade 3 and 8 and shall detect if the child is at risk of developing overweight or obesity. All SHNs in primary health care throughout the country work under the same regulations and national guidelines. The Norwegian national clinical guidelines for prevention, identification, and treatment of overweight and obesity in children and adolescents recommend follow-up or treatment if children or adolescents BMI ≥ iso-BMI 25 [5], after screening for other factors such as family circumstances, PA, diet, and illness, which can have an impact on overweight. Follow-up and treatment by SHNs are optional for the families and focuses on motivating changes in PA and diet, mostly on an individual basis with the young person, with or without the involvement of parents. The present guidelines provide sparse guidance on how to motivate adolescents toward increased PA and how to sustain such motivation. They focus mainly on advice such as walking/cycling to school, taking part actively in physical education (PE), committing the family to engaging in PA together, or exercising vigorously to the point where they sweat and are out of breath [6].

### Recruiting and study participants

To recruit participants, we contacted 12 school health nurses at public health centers in various regions and different-sized municipalities in Norway, to help us come in contact with adolescents for participation. They were asked to recruit adolescents who they had found to have a BMI ≥ iso-BMI 25 at the mandatory measurement of height and weight in grade 8, and where they had initiated follow-up of weight development. Due to SHNs acting as gatekeepers for contact with adolescents, lack of time among SHNs, and other restraining factors, few participants were recruited this way. Therefore, four Healthy Life Centers which are part of the offer in primary health care services were additionally asked to help recruit a purposive sample of adolescents. The adolescents where referred to the Healthy Life Centers as part of the follow-up. The inclusion criteria for participants in this study was that adolescents should be between 13 and 18 years and categorized as having a BMI ≥ iso-BMI 25 by their SHN at the mandatory measurement in grade 8, they should be followed-up in primary health care settings, and in addition the adolescents were excluded if they had other diseases that affected PA participation. In all, 23 adolescents were invited to participate, orally via their SHN or the HLC staff, which informed the adolescents about the study, and invited them to take part in the study by handing out information letter about the nature and aim of the study. The adolescents were part of the study if they met both inclusion criteria and after signing the consent. Two adolescents declined to take part, while 10 never responded. Eleven adolescents participated, five boys and six girls, but as one boy had a BMI < iso-BMI 25, he was excluded from the data.

The sample represented various regions and different-sized municipalities to ensure a diversity of perspectives. Some adolescents (*n* = 7) participated in activity groups for young people with overweight or obesity, as an offer by the HLC in their municipalities. The rest (*n* = 3) were not offered any specific activity for the follow-up of PA. After analyzing interviews, the sample size was decided to hold sufficient information power, based on discussion of the aim of the study, sample specificity, use of established theory, quality of dialogue, and the analysis strategy [28].

### Data collection and procedure

The first author (female PhD-student) conducted the 10 individual in-person interviews included in the analysis with only first author and adolescent present, based on a semi-structured interview guide (supplementary file 1), between February and June 2017. Interviews and field notes were the only data collecting methods used. The interviews were seen as a conversation between the interviewer and interviewee and were conducted in a non-assertive manner, using open-ended questions to explore issues important to the adolescents and to encourage reflection around these issues. The interviewer was not a health worker and had no connection with the adolescents’ school health services but was experienced in conversations with adolescents. The interviewees were assured that information from the interview would not be reported to their SHNs. In advance of the interviews, a pilot interview was conducted, which revealed that small changes were necessary. In view of the challenges in recruiting young people, it did not prove possible to conduct more than one pilot interview. Data from the pilot interview was not included in the results.

The interview guide was developed by the authors for the purposes of the study through an extensive review of studies in the literature of adolescents’ experiences with lifestyle changes and was built on theory arising from this review (supplementary file 1). The main questions were discussed and agreed upon in the research group. Questions were organized around the themes of motivation, preferences, influences, and barriers regarding activity, as well as diet, attitudes, health behaviors, daily life, and experiences regarding measuring weight and follow-up in primary health care. The interviews were conducted at locations according to the young people’s wishes—at home, walking, at the school, or at the local activity center. On two occasions, the first author participated in activity groups with the young people in advance of the interviews. The interviews started with brief information about the basic aims of the study, the interviewer’s reasons and interest in the research topic, the confidential nature of the study, and a statement that the interviewee could at any time withdraw from the study. All interviews were audiotaped and transcribed verbatim. Additional hand-written field notes were taken after each interview, in order to maintain contextual details and non-verbal expressions, for analysis and interpretation of the data. Quotations used in the text have been translated from Norwegian to English. The research group discussed and agreed upon the ethics regarding recontact with the adolescents after completing interviews. Due to the sensitive nature of the theme for these adolescents the transcripts were not returned to participants for comments, but there was a highly focus on explanatory questions during interviews.

### Ethics

The Regional Committees for Medical and Health Research Ethics, More and Romsdal, Sor-Trondelag and Nord-Trondelag, assessed the study but considered that the project did not fall within its mandate (ref. 2016/1800/REK midt). Norwegian Centre for Research Data (NSD) was notified and approved the research project (ref. 50,736). All participants, as well as the parents or guardians of the young people, received an information letter describing the confidential and voluntary nature of the study. All participants and one parent or guardian of the young person signed the information letter by way of giving consent.

### Analysis

For data analysis, systematic text condensation (STC) was used, as it is a descriptive and exploratory method for thematic cross-case analysis, presenting the authentic experience of the participants. This method was chosen for its pragmatic approach and for allowing the researchers to play active, reflective roles in the interpretation of each participant’s account. The first author coded the material using NVivo 11 (QSR International Pty Ltd., Melbourne, Australia), and second author (male associate professor experienced in both quantitative and qualitative methods) read through the material and supplemented the first author’s findings. This process was repeated several times to ensure a broader and more complex understanding of the phenomenon [29]. STC presents a four-step analysis strategy. All steps were conducted for four interviews at a time to manage a sufficient amount of data. Bracketing preconceptions, the researcher read through and listened to the data several times, searching for a general impression and for preliminary themes. The main themes were then discussed by the researchers. The data were examined line by line for meaning units, which were classified and sorted into preliminary codes in an iterative process. The contents in each group were abstracted and sorted into subgroups and thereafter reduced to decontextualized condensates, to systematically re-narrate and summarize the contents of the group. The condensates representing each sub-group amalgamated the contents from the meaning units into one text. During this process, names of code groups were adjusted in line with evolved understanding, and new meaning units from the data were additionally added. Data were reconceptualized by synthesizing the contents of the condensates, and the content of each group was summarized into generalized descriptions. The STC method involves a process of intersubjectivity, reflexivity, and feasibility, while at the same time maintaining a responsible level of methodological rigor [27].

## Results

The adolescents in this study were aged 13–17, all categorized as overweight or obese (BMI ≥ iso-BMI 25) by health professionals (Table 1). Most respondents expressed their gratitude that someone was showing an interest in their situation and were willing to express their experiences with primary health care follow-up and PA, although sometimes they found it a challenge to verbalize their experiences or feelings. Interviews were lasting 42–92 min.

**Table 1:** Characteristics of participants

	No.
**Sex**	
Female	6
Male	4
**Age (years)**	
13	3
14	1
15	1
16	3
17	2
**Municipality size**	
1000–4999	1
5000–9999	1
10,000–29,999	4
30,000–99,999	4
**Area**	
Central Norway	1
Western Norway	1
Eastern Norway	8
**Adolescents with an offer of PA for ow/ob**	
Group based	7
Individual	1
None	2

A systematic and thorough reading of the data (transcripts and field notes) elicited several interesting themes related to the respondents’ experiences of PA in their social network. The social networks presented in this study were identified through the interviews; the PE setting seemed to be a main arena for sharing experiences of PA participation for all respondents in this study. Some respondents reported that they had been participating in organized sports, such as handball or dancing, and some had experiences with this as a supportive social network for engagement in PA as they knew their teammates and coaches for many years, and they felt they were mastering the activity at an acceptable level. Similarly, respondents also identified, in their social networks, some especially important others in their experiences with PA, such as fellow pupils, teachers, coaches, school health nurses, and peers in activity groups. Respondents tended not to emphasize others, such as parents, who are usually mentioned in studies of PA and young people, except some adolescents expressing support from parents for being physical active. The analysis revealed barriers to and facilitators of advice for increasing their PA level experienced by these respondents, and both were analyzed in order to construct the big picture. Among those who already had an existing network with friends with whom to be active and those who had been introduced to a social network for PA through activity groups for adolescents who are overweight and obese, respondents described a social network for PA as a perceived facilitation. Most respondents in this study reported that the recommendations they received from SHNs largely corresponded with advice for increased PA listed in the national clinical guidelines for the prevention, identification, and treatment of overweight and obesity in children and adolescents [6].

From the data, several interesting experiences from the respondents emerged, which were organized under three main themes, with two sub-themes each. Sub-themes describe both barriers to and facilitators for participating in PA, as described by the young people (Table 2). The results are presented in the same order as in Table 2, each main theme is presented with two sub-themes, each representing adolescents experienced barriers first and then facilitators.

**Table 2:** Main themes, sub-themes and meaning units

Main theme	Sub-theme	Example of meaning unit
**Interests**	*Barrier* **Just something to do**	*“It has always been about changing diet, cycling, exercising and all that. In the end I was shit tired.”*
	*Facilitator* **Variety of activities**	*“It is fun to change activities and not just do one thing. That is part of it, not taking part only in basketball or indoor bandy.”*
**Peers/Others**	*Barrier* **Not good enough**	*“It wasn’t just that you felt you couldn’t do it. Someone told you “You can’t do this”, without actually saying it in words.”*
	*Facilitator* **Be like others**	*“When I run up a flight of stairs, I do not get tired. I feel that the body is healthier and happier. I can do the same as others do.”*
**Situation**	*Barrier* **No understanding**	*“I hate swimming with others. That is some of the most difficult. No one understands that I don’t think it is any fun. Everyone just tries to push me to join.”*
	*Facilitator* **Good relationships**	*“I went there, and everyone was just very nice. I met those who were there, and I just wanted to exercise there.”*

### Interests

#### Just something to do

The respondents felt the SHNs were preoccupied with just finding activities for them to engage in, such as cycling, jogging, walking, and soccer. Many respondents were tired of always being encouraged to participate in PA and felt that it was just about finding something for them to do. They did not have any particular interest in doing these activities and felt that the SHNs needed more background information about them.

*“It’s soccer here, but I don’t want to start with soccer. But I don’t think there is that much more to do here…”*

(I–9, boy attending activity group, age 13)

One respondent said that the only thing she experienced was the pressure to just do something.

*“There will not be much out of it. So I did not get anything out of it other than that I felt pressured to do something.”*

*(I–11, girl not attending activity group, age 16)*

Another girl, from a municipality without any activity group for adolescents who are overweight or obese, had several experiences with SHNs that didn’t have any suitable activity to offer her, and she felt they were just throwing out suggestions.

*“They are just saying: “You can try this, and you can try that.” And that’s very like..”*

(I–6, girl not attending activity group, age 17)

Several expressed that suggestions of activities that did not meet their interest did not lead to physical activity participation.

*“It doesn’t help me, because I don’t like things like this. When she suggested cycling to school. I don’t want to do things like that. I don’t bother, to be quite honest.”*

(I–11, girl not attending activity group, age 16)

#### Variety in activities

The respondents felt that variety and influence in PA promoted their enjoyment. Some reported that when previously participating in organized sports, they had become bored after a short while because they were doing the same exercises at every session and most did not have a special interest in any one particular sport.

*“It is fun to change activities and not just do one thing. That is part of it, not taking part only in basketball or indoor bandy. I attended three soccer practices or something, before I started to find it boring.*”

(I–5, girl attending activity group, age 17)

Respondents introduced to activity groups for young people with overweight or obesity reported, on the other hand, that the activity groups were settings where they had fun and that provided activities that were varied, easy to perform, and that properly tired them out.

*“Because they always ask us what we want to do next session and the one after that. And we always have suggestions, and we get to do what we’ve suggested at the end of the session and things like that. And we always have activities that are quite simple to do, but we also get tired out. ( … ) It’s fun, social, and physically demanding. And it’s strenuous. We get something out of it, because it’s only one hour.”*

(I–3, girl attending activity group, age 16)

This is in contrast to physical education (PE) classes in school, which they often described in the same way as organized sports, namely, having little variety and choice.

*“I think it was the class environment. And the teachers, what we did and things like that. There wasn’t any variety, I think.”*

(I–2, boy attending activity group, age 17)

### Peers/others

#### Not good enough

PA, in the form of PE with classmates, was perceived as embarrassing because of the feeling of always coming last and getting exhausted in front of others. This was considered a huge failure and led to their avoidance of PE or certain activities in the PE setting. In addition, they were afraid of being laughed at by their classmates.

*“It is embarrassing to always come last. It takes a lot of effort and motivation to say: ‘Yes, today I am going to PE to actually jog.’ Behind the others.”*

(I–11, girl not attending activity group, age 16)

*“I have noticed that I am sometimes out of breath while the others just stand there with their mouths closed. [PE class]”*

(I–7, girl attending activity group, age 13)

Most respondents had negative experiences with PE and felt the classes are designed to cater for pupils with skills and talent. They felt that teachers compare them to the better pupils, but they also compare themselves to classmates. Several respondents reported that they were not capable of doing all the activities in PE, as they were in actual physical pain. Classmates believed them to be inventing excuses about being in pain when they withdrew from an activity.

*“I can’t manage to participate in everything. I can’t run for 45 minutes, as all the others manage to do, because it makes me hurt all over.”*

(I–6, girl not attending activity group, age 17)

They also reported that they didn’t feel competent or motivated to take part in competitive sports in generalized settings.

*“I would like to play handball, but I don’t want to be in a competitive team as soon as I start. But it’s impossible to not start in a competitive team here.”*

(I–3, girl attending activity group, age 16)

#### Be like the others

Several respondents reported that, since starting exercise, mainly in activity groups for young people who are overweight and obese, they were more capable of managing situations with friends during school and leisure time. They expressed pride in exercising and feeling good, knowing that they were doing something beneficial for themselves. Even though many disliked soccer in PE and in organized sports, they liked it in activity groups, because there was no “soccer person” there.

*“I wasn’t a soccer type of person when I was growing up. I have never liked it. But I like to play it here [activity group]. … There are no soccer aces here.”*

(I–2, boy attending activity group, age 17)

They appreciate PA groups, where they can do the exercises and feel the benefits to their health.

*“We do have a thing called wall-sit, a lactic acid thing. It was a nightmare at the beginning. I could barely do 10 seconds, but now I manage almost all 30 seconds. … I have noticed huge differences in how much I am able to manage.”*

(I–2, boy attending activity group, age 17)

*“Then I feel tired and good [After a session at activity group]. I feel that I have done something good for myself.”*

(I–3, girl attending activity group, age 16)

One boy said that he often felt happy after exercise because it was fun, and he found he could do what his friends could do.

*“For example, when I am with friends and they are doing something, and I am able to do the same as them. For example, jump over a fence. I couldn’t do that earlier, because it was hard for me to push myself up. Because then I was carrying a lot of extra weight and I had no muscles. I didn’t feel good when everybody jumped over the fence and then they went on, and I had to take the long way round. I didn’t like that.”* (I–4, boy attending activity group, age 15)

The feeling of improvement in health and managing to do the same things as the others as a result of starting to exercise was also perceived as a victory among respondents who did not attend activity groups.

*“When I run up the stairs, I don’t get exhausted now. I feel my body is healthier and in better shape. I can do the same things as other people do.”*

(I–11, girl not attending activity group, age 16)

### Situation

#### No understanding

Many respondents report that PE teachers lack sympathy and understanding. They perceive most PE teachers as not having a plan for alternative exercises for students with overweight or obesity and expecting the same performance from everyone in the class.

*“If we were supposed to run [in PE class], he didn’t think about me. He just said like… I had to go over to him and say, ‘You know I can’t run.’ And then he said, ‘You can go to the weights room.’ But he didn’t have a plan. He didn’t have an alternative plan.”*

(I–6, girl not attending activity group, age 17)

*“Teachers in lower secondary school saw the whole class as one pupil. For everyone to be good, they had to do [exercises or activities] exactly the same as everyone else.”* (I–2, boy attending activity group, age 17)

One girl spoke about not being able to run with the rest of the class, how her classmates did not understand, and how her teacher told her to just keep on doing it. The respondents felt that both classmates and teachers thought they were just making excuses.

*“I thought it was hard. I felt I couldn’t breathe. I remember it was awful, because people didn’t understand why I couldn’t do it. They were all like that, and the PE teacher said that I should just keep on trying. Some PE teachers could be very like that... Having no understanding.”*

(I–11, girl not attending activity group, age 16)

*“ … it gives me physical pain all over. And there is no one in the class that understands that. They think I’m making excuses. And that’s different from what I’m really doing. So, it’s more like “Oh, there she is, sitting down again. She couldn’t manage to do this.’ It’s so wrong. They don’t understand, in a way.”*

(I–6, girl not attending activity group, age 17)

#### Good relationships

The respondents reported that a social network where they felt equal to other participants made PA fun, enjoyable, and a positive experience. Respondents without an existing social network, who were offered an activity group for young people with overweight and obesity, felt positive about PA in this setting.

*“When I started [activity group], I thought it would be really boring and so on. I thought it would be no fun here. I’ve been proven wrong, you might say. … It’s the coaches, of course. And the others in the group. It has always been some ‘non-persons’, a bit negative. But the positive ones always make up for the negative ones. I’m very happy that I have friends here and all that. People I come along with are a lot of fun to exercise with.”*

(I–2, boy attending activity group, age 17)

They felt they had many friends in the group, and it was important to them that the other members were also not so good at sport. The importance of being in a group where everyone had a weight issue was underlined. One girl said that it is important to meet someone who thinks the same as you and emphasized the fact that they are all alike as the reason to continue in the activity group.

*“It’s not that I think it’s good that everyone has some excess fat, but it does something for you when you realize that everybody has some. Because then no one feels any bigger than the others.”*

(I–3, girl attending activity group, age 16)

The respondents perceived it as safe when everyone had a weight issue.

*“Because then you feel safer. … for example, most of us are a bit stocky. … Except one... and if there had been several… and if you exercise, they start laughing if you do something wrong or something. And if there had been strangers present, then they would have started talking … They’d say bad things about you and things like that.”* (I–9, boy attending activity group, age 13)

Some respondents that had been participating in organized sports over several years also reported this as a place where they had a supporting network for PA and where they felt equal.

“*I feel I’m on a good team where I can have fun with other people. The fact that I run a bit slower sometimes is okay, because others do it as well. It’s not everyone who can run fast. So it’s fun, and I think that is very nice. And then I have fun when I’m doing an activity.”*

(I–11, girl not attending activity group, age 16)

Some PE teachers were perceived as caring when they had alternative exercises available for those who could not manage an activity. Coaches in activity groups were perceived as personable, funny, and happy to see and greet them.

*“I am the kind of person that doesn’t want to test new things because I’m afraid of meeting someone I know or someone that doesn’t like me. But here I always know who I will meet, and there are always nice people who say ‘hello’ and that is always nice.”* (I–3, girl attending activity group, age 17)

Respondents feel it is important that they are familiar with the coaches and that the coaches are nice to them.

*“And there are coaches who are so funny and nice and bubbly and happy to see us and they always says ‘hello’. And they always have a plan for the session, so I know what I’m supposed to do.”*

(I–2, boy attending activity group, age 17)

## Discussion

This study aims to provide insight into how adolescents who are overweight or obese and receive follow-up of weight management in primary health care, experience barriers to and facilitators to engaging in physical activity within their social networks.

### Experience of physical activity in the young people’s social networks

The present study reveals that the respondents place a strong emphasis on a social network where they feel equal to others if they are to engage in PA. Other research has suggested that forming groups that consist of similarly young people who are overweight can contribute to an increased PA level and help modify their negative perceptions of such activity [18]. In this study, most of the respondents described a supporting social network with young people in the same situation as themselves as an important facilitator for PA participation. A setting where they feel equal to others makes them feel safe and competent. In agreement with this study, Reece et al. [30] found that adolescents who are overweight engaged in treatment and interacting with peers in a similar situation to themselves perceived this as socially supportive and made PA enjoyable. Their data indicated that the group arrangement may have been one of the first times the adolescents had experienced being physically active with peers in a safe and supportive environment [30]. In the present study, adolescents introduced to activity groups for the overweight and obese reported similar experiences. Other qualitative research also finds that adolescents who are obese describe a group with others in the same situation as an arena in which to share experiences, get peer support, and perhaps join in activities together [13]. A few adolescents also expressed support from parents for engaging in physical activity, this could be of importance for this group of young people as research has shown that activity-related support from parents may facilitate PA participation in youth at risk of inactivity [15].

The experience of PA in PE at school contrasts with the experience of PA in activity groups. In the PE setting, many of the respondents in the present study said they do not feel competent compared to other pupils. They perceive the PE setting as a place where they feel embarrassed and vulnerable and where their physical capabilities and appearance make social interaction with peers difficult. For some of the respondents, PE is the only social setting for organized PA and perhaps an essential setting in which to form perceptions of what it is like to take part in PA [26]. Negative experiences from the PE setting could partly explain why some of the respondents do not manage to follow up on SHNs’ recommendations for more PA participation in social settings such as organized sports.

In line with other studies [11, 31, 32], the present findings reveal a dislike of being visible to others in PE. PE seems to be perceived by the respondents as a form of competitive activity, with expectations of certain skills that they are unable to fulfill. They are afraid of being spoken ill of or laughed at and feel embarrassed about their own perceived lack of capability. This agrees with research [11, 31, 33] that indicates adolescents who are overweight and obese to be at increased risk of victimization and bullying. Peer victimization is negatively related to PA in children and adolescents who are overweight and at-risk-for-overweight [11, 31, 33]. Reducing body-related concerns, such as others seeing their body while in activity, may serve as PA intervention points [32]. It is important for SHNs to be aware of the increased risk to this group of young people, as the national guidelines recommend increased participation in just these vulnerable arenas [6].

A few respondents who had taken part in organized sports for several years perceived this as a positive social setting for PA, mainly because they found it supportive and safe. They had known their teammates and the coaches for many years, and they felt they were mastering the expected skills to enable them to participate. The present findings are supported by Sundar et al. [26] on young people who are overweight or obese experiences with PA. They also found that long acquaintance with teammates and the feeling of mastering the activity were important factors encouraging participation in organized sports. According to SDT, it is important for participants not to be over-challenged but helped to experience mastery. In addition, the sense of being respected, understood, and cared for is essential for them to experience a connection and trust that further allow the internalization of a health-promoting behavior [7].

### Barriers to and facilitators for engaging in PA

Several respondents described a lack of an understanding of their interests as a barrier to engaging in the recommended PA. Some respondents felt that the SHNs did not know them well enough to make appropriate recommendations and had too little background information. They felt that the SHNs’ advice on PA did not meet their interests or needs. The result was a feeling of pressure just to *do something*, which, far from increasing their motivation to participate in PA, undermined it [7]. Pressure from SHNs places them in a position of having their motivation controlled, and this can have a depressing effect on the quality of their output [34]. In municipalities where there was no activity available specifically for this group of adolescents, the respondents in this study felt that the SHNs did not really have anything to offer them. The consequence was that adolescents lost interest in taking part in any activity. Most of the respondents were keen to engage in varied activities and reported that having an influence in choosing them was something they experienced as positive. Mandatory PE and organized sports were described by most of the respondents as affording little variety in activities and little adaptation to those who were overweight. Hence, they were inclined to perceive organized sport in particular as inaccessible, which could then limit their opportunities to participate [26].

Regarding one of the main themes, *peers/others*, the respondents described their main barrier as the feeling of not being good enough. This feeling is related both to their own expectations and to perceived expectations from peers and teachers. Competence is seen as a fundamental element in motivated action, and as one of the three basic psychological needs that must be satisfied in order to facilitate growth, integrity, and well-being, as described in SDT. In contexts where challenges are too difficult, negative feedback is prevalent. Similarly, when the feeling of mastering the activity or effectiveness is weakened by interpersonal factors such as personal criticism and social comparisons, the feeling of competence decreases [34]. On the other hand, the respondents in this study describe managing activities, getting better at different exercises and equaling the performance of their peers as important. Participation in activity groups seem to offer these experiences. Feelings of satisfaction, competence, and mastery enhance motivation for further PA participation.

Another barrier the respondents mentioned was the perceived lack of sympathy and understanding of their situation from others. By contrast, a social network in which they are on the same level as others and therefore experience mastery in the same way is important. Furthermore, good relations with peers, teachers, and coaches increase motivation to maintain PA. The latter applies to both PE at high school (in vocational education), where the pupils are “*not so sporty*,” and to organized sports or activity groups for adolescents who are overweight or obese. In regard to relationships, the activity groups differ from PE at school and organized sports. Those attending activity groups perceive the staff as understanding their perspectives, providing choices, being supportive, and encouraging more autonomy than most PE teachers. However, both activity groups and organized sports (when this is perceived as a network with supportive peers and coaches) seem to be autonomy-supportive environments. Authorities (i.e., coaches) take the perspectives of individuals into consideration, which satisfies the need for relatedness and enhances a sense of belonging [23, 34, 35]. This in turn has been shown to predict more autonomous motivation, a lower calorie intake, and increased PA [24]. In addition to offering choice, research shows that a neutral language must be used during interpersonal communication [34].

The respondents describe PA in some settings as self-rewarding. They speak of “doing something good for myself” and “it is fun.” Thus, they may endorse or identify with the value of the behavior, which is associated with improved maintenance and transfer of behavior change [7]. This indicates that forming activity groups for young people who are overweight or obese who lack a supportive social network for PA could encourage more autonomous motivation through identified regulation. Respondents introduced to activity groups for young people who are overweight or obese emphasized this as a way into a social network. According to SDT, a social context that fulfills basic psychological needs, such as activity groups in this study, enables people to internalize and integrate social values and practices and thus enhance their social effectiveness and connectedness [36]. By contrast, the lack of a social network seems to make it more difficult for these respondents to adopt and maintain health-promoting habits that include PA. In line with Ng et al. (2014) and Ng et al. (2013), the present findings indicate the strong impact of important others [23, 24].

SDT assumes that even motivation that is of a more controlled form can be internalized and transformed into more autonomous motivation, if the three basic psychological needs (autonomy, competence, and relatedness) are supported by the environment [20, 35]. This seems to be the case in the present study, where respondents were first introduced to the activity groups by SHNs in a primary health care setting, and therefore for more controlled reasons. After attending the groups, they describe motivation based on appreciation of the variety of activities and their influence on the activities that are offered, how well they manage the activities, and the good relationships they enjoy. Other research has also reported increased engagement and satisfaction in PA among young people when there is a variety of activities available [37]. According to SDT, satisfaction of the three psychological needs is a determinant of people’s engagement in and maintenance of behavior change [20, 38]. The importance of more autonomous motivation to undertake exercise to promote a long-lasting active lifestyle is also shown in research on children with overweight [39]. SHNs recommendations to increase PA among adolescents who lack a social network for PA seems to reinforce their negative association with PA and hence undermine their motivation to participate in PA. Activity guidance to this group of young people therefore should focus on establishing a supportive social network for PA which address autonomy, competence and relatedness. This is to foster motivation and engagement for activity, as described in SDT [20]. One important finding in this study is the adolescents’ negative experiences with PA in PE classes. This is an important finding due to the aforementioned unique position PE have, for some, as the only social setting for organized PA and therefore an essential setting in which to form perceptions of what it is like to take part in PA [26]. As stimulating lifelong joy of movement and a physically active lifestyle based on one’s own preconditions is a central value of Physical Education in Norway [40], it could be of value to mitigate the discomfort that adolescents who are overweight and obese describe when participating in these classes. PE teachers should therefore also focus on establishing a supportive environment that address autonomy, competence and relatedness for PE to serve as a motivator and not as a barrier for a physical active lifestyle. Results from this study indicate that adolescents that have participated in especially activity groups for adolescents with overweight or obesity emphasize an environment for physical activity that offer a variety of activities without the competitive nature, activities they feel competent to participate in, and a supportive environment where they feel safe.

#### Limitations

This article does not include the perspectives of those who were invited to participate in activity groups for young people who are overweight or obese and either chose not to participate or who quit at some juncture, because of the difficulty of ascertaining their views. It may be that the respondents who choose to participate in activity groups are already more motivated to make changes in their PA than non-participants. A comparison of the experiences of non-participating adolescents with those of participants in activity groups in this study could have added important knowledge to the perceived differences [41]. However, in spite of this limitation, it can be asserted that the respondents have provided true opinions and that the findings add important knowledge about young people with overweight’s experience of and attitudes to PA. Several of the findings are supported by other research, and studies of young people who are obese also show that there is a discrepancy between what they say will be helpful to them and what results show is actually useful to them in the maintenance of interventions [42].

The strength of this study on this point is that the respondents expressed experiences during or after follow-up in primary health care and that those participating in activity groups expressed their experiences during participation. We believe the qualitative nature of this study is a strength, since the interviews provide in-depth insight into how the adolescents experience their own situation. A strength of this study is that the participants represented various regions and different-sized municipalities in Norway and the sample therefore may ensure a diversity of perspectives, in addition the sample comprised almost as many male participants (*n* = 4) as female participants (*n* = 6). Further there is a lack of studies that focus on experiences from adolescents outside of study-interventions or specialist health care, and such experiences may help expand health workers in primary health care settings to better understand the adolescents situations and make them able to match interventions to the needs and expectations of these youngsters.

## Conclusion

The aim of this study was to explore adolescents’, who receive follow-up of weight management in primary health care, experienced barriers to and facilitators for engaging in physical activity within their social networks. The adolescents expressed that the feeling of SHNs being occupied in just find something to do, the feeling of not being good enough and lack of an understanding for their situation as barriers for PA participation. Variety of activities, the feeling of being like the others and good relationships was expressed as facilitators for engagement and participation in PA.

By provide insight into the barriers to and facilitators for engagement and participation in PA, this study contributes to improved understanding of how SHNs’ advice is experienced by adolescents who are overweight. Further, the answers from the respondents in this study point to the value of forming activity groups that consists of similar young people with overweight, rather than increased participation in ordinary organized sports. This is especially important for young people who do not already have a social network for PA participation. Implications for practice based on the study findings therefore emphasize that SHNs should map the conditions for PA in the adolescent’s social network before any advice on increased PA engagement is given. For those adolescents that lack a social network for PA, for School health Services to establish a social network of adolescents in the same situation is found to facilitate an increase in the PA level in these individuals. Interventions based on advice in the national clinical guidelines in Norway [4] to increase PA among adolescents who lack a social network for PA seems to reinforce their negative association with such activity. Given the present findings, future research should focus on SHNs’ experiences with adolescents who are overweight or obese barriers to and facilitators for participation in PA.

## Supplementary Information

**Additional file 1.** Interview guide – Adolescent.

## Data Availability

The datasets generated and analyzed during the current study are not publicly available, due to participant confidentiality, but are available from the corresponding author on reasonable request.

## Abbreviations

PA: Physical activity
SHN: School health nurses
PE: Physical education
HLC: Healthy Life Centers
SDT: Self-determination Theory
STC: Systematic text condensation
