# Supplementary material for: Adolescents who are overweight or obese - the relevance of a social network to engaging in physical activity: a qualitative study
Source: BMC Public Health. 2021 Apr 9;21:701. doi: 10.1186/s12889-021-10727-7 (PMC8034089; doi:10.1186/s12889-021-10727-7)
Supplement: Supplementary file 1 — Additional file 1. Interview guide – Adolescent. [file 12889_2021_10727_MOESM1_ESM.docx]

**Interview guide** – *Adolescent*

**Aim:** Examine young people’s experiences of barriers and facilitators of healthy lifestyles

**Problem areas:**

- Activity habits
- Attitudes – lifestyle and conditions for change
- Health behavior
- Others attitudes/influence
- Experiences with weight measuring
- Experiences of follow-up
- Future

**Form**

*1. Framework*

- Informal conversation
- Information about the project and the problem

*2. Entrance and motivation for participation*

- Transitional issues

*3. Focusing*

- Key issues
- Focus on specific events (“what do you mean?”, “Can you clarify?”, “How did you experience..?”, exemplification, vivid answers, ask for episodes, etc.)
- Follow-up questions (perception of the meaning content can provide a starting point for follow-up questions)

*4. Looking back*

- Summary
- Clarification

| Research area | Interview question |
| --- | --- |
| Motivation for participation | Why did you agree to participate in this study?   - What do you think may be the reason why someone has said they do not want to participate? |
| Activity habits – preferences, motivation, influence and barriers | Tell about a regular day – what do you do?   - What do you think it means to have a healthy lifestyle? The ideal. - How is your lifestyle in relation to this? - Where do you learn about lifestyle? Home/school/peers/media? Is there any difference? - Do you do any activities or sports? Why? - What kind of activities do you like/dislike? - How do you experience PE? Activities/fellow students/teachers/locker room? - Tell about activity during school hours/leisure time. - Is there an offer in the local community that fits your interest? - What do you do with friends? - Tell about the activity to the rest of your family. - Tell about why you sometimes are not physically active. - Do you think your activity is influenced by anyone? Friends/parents/media/SHN? - Who motivates you to be physically active? How? School/home/leisure time. - What would have helped you to be more active? |
| Attitudes – lifestyle and conditions for change | You go to follow-up because you are going to try to change your lifestyle a little/get control of your weight development. I would like to ask you a little about lifestyle and your experiences with this.   - Do you need to change your lifestyle? Why/why not? - What made you understand (that someone thought) you should change your lifestyle? Concrete event. - Who/what made you aware of, and how? - How do you find that this is true for you? - Concrete example of something you think you should change. - What is challenging about this? - What do you do to have a healthy lifestyle? |
| Health behavior – barriers and facilitators | - What can you do to help yourself to be healthy? What can others do? Example - Is there a difference in what it is like to make healthy choices in different situations? At school/home/leisure time/with friends/alone/in your environment? How? - What makes it easier/more difficult? - Have you tried to do something with your lifestyle earlier? |
| Others attitudes/influence | - Do you feel that parents/peers/siblings/teachers/others are interested in physical activity? How? - What kind of attitudes are there in your social network when it comes to lifestyle? - What kind of activity is important in your social network? - Who in your life thinks you should change your lifestyle? (Teachers/family/friends/media/SHN?) Why? - What do you experience that others think about lifestyle/body? (Teachers/family/friends/media/SHN/doctor?) Concrete example. - Does this have anything to do with your lifestyle? - Who is talking to you about lifestyle/overweight/body? When/where/why? |
| Experiences with weight measuring | Are you weighed and measured by a nurse? When?  Tell about the last time you weighed and measured yourself at the school health service.   - How did you experience that the SHN talked to you about lifestyle and health? - How do you experience the SHN looking at you as a person? - What do you think about weighing and measuring with the SHN? - Did it have anything to do with the fact that you were told that you should receive follow-up/help/advice? How? |
| Experiences of follow-up | Tell about what happens when you are with the SHN – last time   - What does the SHN help you with? - What did you experience as the reason why the SHN thought you should change your lifestyle? - If overweight – how does the SHN explain this? - What do you experience as the reason why the SHN thinks it is wise for you to change your lifestyle? - Do you experience that the SHN knows what she is talking about? - What do you expect to get out of follow-up? - How do you feel that your parents are involved in the conversation? How important is it? What would it have been like if they had not participated? - How important is the SHN for you to change your lifestyle? - What do you think the SHN could have done differently to help you?   Tell about a guidance interview, what made you follow/not follow the advices?   - What motivates you to follow the advice? (What’s going well?) - What demotivates you? (What is difficult?) - Are there situations, places, people that make it more difficult to follow the advices? - What kind of follow-up do you think would have been the best for you? - What do you want from someone who should help you change your lifestyle? |
| Future | - How do you see your opportunities regarding a change in lifestyle? - Who/What do you think might help for you to change lifestyle? - How is your motivation regarding having healthier lifestyle? - How do you see your lifestyle in five years? - What do you think should be the role of the SHN when young people need to change their lifestyle? - What would be your advice to politicians that could make it easier for young people to live healthy lives? |
| Demographic issues | Age  BMI  Parents occupation |
| Looking back | Clarification   - Summary - Clarify misunderstandings, ask if you have understood correctly if something is unclear. - Is there anything else you want to add? |
